# The potential role of SARS‐CoV‐2 infection in acute coronary syndrome and type 2 myocardial infarction (T2MI): Intertwining spread

**DOI:** 10.1002/iid3.798

**Published:** 2023-03-17

**Authors:** Aseel Awad Alsaidan, Hayder M. Al‐Kuraishy, Ali I. Al‐Gareeb, Athanasios Alexiou, Marios Papadakis, Khalid Adel Alsayed, Hebatallah M. Saad, Gaber El‐Saber Batiha

**Affiliations:** ^1^ Department of Family and Community Medicine, College of Medicine Jouf University Sakaka Saudi Arabia; ^2^ Department of Clinical Pharmacology and Medicine, College of Medicine ALmustansiriyia University Baghdad Iraq; ^3^ Department of Science and Engineering Novel Global Community Educational Foundation Hebersham New South Wales Australia; ^4^ Department of Research and Development AFNP Med Wien Austria; ^5^ Department of Surgery II, University Hospital Witten‐Herdecke, Heusnerstrasse 40 University of Witten‐Herdecke Wuppertal Germany; ^6^ Department of Family and Community Medicine Security Forces Hospital Program Riyadh Saudi Arabia; ^7^ Department of Pathology, Faculty of Veterinary Medicine Matrouh University Matrouh Egypt; ^8^ Department of Pharmacology and Therapeutics, Faculty of Veterinary Medicine Damanhour University AlBeheira Egypt

**Keywords:** acute coronary syndrome, acute myocardial ischemia, arrhythmias, atherosclerotic plaques, COVID‐19, SARS‐Cov‐2 infection

## Abstract

Coronavirus disease 2019 (COVID‐19) is a novel pandemic caused by severe acute respiratory syndrome coronavirus 2 (SARS‐CoV‐2). It has been shown that SARS‐CoV‐2 infection‐induced inflammatory and oxidative stress and associated endothelial dysfunction may lead to the development of acute coronary syndrome (ACS). Therefore, this review aimed to ascertain the link between severe SARS‐CoV‐2 infection and ACS. ACS is a spectrum of acute myocardial ischemia due to a sudden decrease in coronary blood flow, ranging from unstable angina to myocardial infarction (MI). Primary or type 1 MI (T1MI) is mainly caused by coronary plaque rupture and/or erosion with subsequent occlusive thrombosis. Secondary or type 2 MI (T2MI) is due to cardiac and systemic disorders without acute coronary atherothrombotic disruption. Acute SARS‐CoV‐2 infection is linked with the development of nonobstructive coronary disorders such as coronary vasospasm, dilated cardiomyopathy, myocardial fibrosis, and myocarditis. Furthermore, SARS‐CoV‐2 infection is associated with systemic inflammation that might affect coronary atherosclerotic plaque stability through augmentation of cardiac preload and afterload. Nevertheless, major coronary vessels with atherosclerotic plaques develop minor inflammation during COVID‐19 since coronary arteries are not initially and primarily targeted by SARS‐CoV‐2 due to low expression of angiotensin‐converting enzyme 2 in coronary vessels. In conclusion, SARS‐CoV‐2 infection through hypercytokinemia, direct cardiomyocyte injury, and dysregulation of the renin‐angiotensin system may aggravate underlying ACS or cause new‐onset T2MI. As well, arrhythmias induced by anti‐COVID‐19 medications could worsen underlying ACS.

## Graphical abstract

SARS‐CoV‐2 infection through hypercytokinemia, direct cardiomyocyte injury, and dysregulation of the renin‐angiotensin system may aggravate acute coronary syndrome (ACS) or cause new‐onset type 2 myocardial infarction. As well, arrhythmias induced by anti‐COVID‐19 medications could worsen ACS.

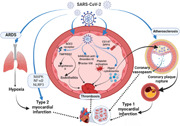

## 1 INTRODUCTION

Coronavirus disease 2019 (COVID‐19) is a new international pandemic caused by a novel severe acute respiratory syndrome coronavirus 2 (SARS‐CoV‐2), which was initially documented in Wuhan, China agreeing to the preliminary epidemiological reports.^1^, ^2^ COVID‐19 affects millions of people, and up to late April 2022, the number of infected people reaches more than 550 million. However, most of the affected subjects are asymptomatic or exist with a mild presentation in 80%; nevertheless, 15%–20% of infected patients necessitate to be hospitalized due to the progress of acute lung injury (ALI) and in severe cases due to acute respiratory distress syndrome (ARDS).^3^, ^4^ Critical COVID‐19 patients require mechanical ventilation and intensive monitoring in the intensive care unit (ICU). Severe and critical COVID‐19 cases are associated with the development of hyper‐inflammations and cytokine storm (CS).^5^, ^6^

The fundamental causes of inflammatory and immunological instabilities in patients with critical COVID‐19 are linked to the overestimating T cells and macrophages with the succeeding discharge of an enormous sum of proinflammatory cytokines such as interleukins (ILs) and chemokines.^7^, ^8^, ^9^ Notably, IL‐6, IL1β, IL‐8, and tumor necrosis factor‐alpha (TNF‐α) through severe SARS‐CoV‐2 infections are related to the progress of ALI/ARDS and multiorgan failure (MOF).^10^, ^11^ Also, one of the most predictable entry‐point of SARS‐CoV‐2 is an angiotensin‐converting enzyme 2 (ACE2). The interaction between SARS‐CoV‐2 and ACE2 leads to a significant reduction of this anti‐inflammatory receptor. ACE2 is intricate in the control of the renin‐angiotensin system (RAS) through metabolism and conversion of inflammatory vasoconstrictor angiotensin II (Ang II) into vasodilator anti‐inflammatory Ang 1‐7 and Ang 1‐9.^12^, ^13^ Therefore, the downregulation of ACE2 and elevation of circulating AngII during SARS‐CoV‐2 infection might be the probable mechanism behind the induction of inflammatory disturbances.^14^, ^15^

It has been shown that severe SARS‐CoV‐2 infection due to the development of inflammatory and oxidative stress and associated endothelial dysfunction may lead to the development of acute coronary syndrome (ACS).^16^, ^17^ Therefore, this review aimed to revise the published papers and ascertain the link between severe SARS‐CoV‐2 infection and ACS.

## 2 ACS OVERVIEW

ACS is a spectrum of acute myocardial ischemia due to a sudden decrease in coronary blood flow, ranging from unstable angina to myocardial infarction (MI), which is either ST‐elevation MI (ST‐MI) to non‐ST‐elevation MI (NST‐MI).^18^, ^19^ ST‐MI reflects a total reduction of coronary blood flow caused by complete occlusion of coronary vessels; however, NST‐MI reflects a partial reduction of coronary blood flow caused by partial occlusion of coronary vessels similar to unstable angina.^20^, ^21^ Acute MI is defined according to the last fourth definition as the presence of acute MI detected by abnormal cardiac biomarkers in the setting of evidence of acute myocardial ischemia.^22^ Acute MI is classified based on ST‐segment elevation on the ECG and is further classified into six types: infarction due to coronary atherothrombosis (type 1), infarction due to a supply‐demand mismatch that is not the result of acute atherothrombosis (type 2), infarction causing sudden death without the opportunity for biomarker or ECG confirmation (type 3), infarction related to a percutaneous coronary intervention (PCI) (type 4a), infarction related to thrombosis of a coronary stent (type 4b), and infarction related to coronary‐artery bypass grafting (CABG) (type 5).^23^, ^24^

ACS remains widely prevalent and still is the top cause of death in people over 35 years of age. ACS is associated with very high morbidity and mortality and is best managed by an interprofessional team that includes the emergency department physician, cardiologist, internist, pharmacist, and primary caregivers. The condition is primarily managed by the cardiologist but the prevention is managed by the primary care provider and nurse practitioner. ACS affects about 15.5 million in the United States. The American Heart Association estimates a person has a heart attack every 41 s.^25^ A recent systematic review and meta‐analysis showed that the prevalence of ACS differs across geographical regions, highest in Australia (21.54%) and lowest in Asia (8.59%). No significant difference in prevalence was found between high‐income and upper‐middle‐income countries.^26^

Recent research in the last decade has altered our view of ACS from a mere lipid deposition to an inflammatory disease; from ACS exclusively due to plaque rupture to the novel definitions of plaque erosion or calcified nodule; from the notion of a superimposed thrombus with necessary lethal consequences to the concept of healed plaques and thrombus contributing to plaque progression.^26^, ^27^ The risk factors for the development of ACS include hypercholesterolemia, hypertension, diabetes mellitus, smoking, and obesity. Obese patients manifest ACS at a younger age.^28^ Accurate diagnosis and early risk stratification are important in guiding treatment and predicting the prognosis of patients with ACS. Recently, attention has been focused on the potential role of plasma markers of inflammation as risk predictors in ACS. Of these markers are C‐reactive protein (CRP) and white blood cell (WBC).^28^

The underlying mechanisms of ACS are reduction of myocardial perfusion (coronary embolism, microvascular dysfunction, coronary vasospasm, severe anemia, severe hypotension, and shock) or increased myocardial oxygen demand (severe hypertension and severe tachyarrhythmia) as well as other systemic disorders such as pulmonary embolism, acute infection, pulmonary hypertension, myocarditis, and stroke.^27^, ^29^, ^30^, ^31^ Therefore, in addition to well‐known risk factors for the development of ACS, inflammatory and oxidative stress complications can contribute to the pathogenesis of ACS.

## 3 RESPIRATORY INFECTIONS AND RISK OF ACS

ACS and T2MI are commonly recognized by atypical presentations such as dyspnea and hypotension in critically ill patients, electrocardiographic (ECG) change mainly in ST‐segment and Q waves, and routine assessment of cardiac troponin (cTn).^32^, ^33^ It has been reported that acute sepsis and respiratory infections are associated with the development of ACS and T2MI through the progression of respiratory failure and severe hypoxemia that reduces myocardial oxygen supply.^34^, ^35^ Besides, sympathetic activation during acute respiratory failure triggers myocardial contractility and oxygen demand.^36^, ^37^ However, T2MI in severely affected patients may be unrecognized, mainly in diabetic patients with autonomic neuropathy.^38^, ^39^ Furthermore, it has been shown that some patients that died from respiratory failure had MI in postmortem studies.^40^, ^41^

Furthermore, respiratory tract infections provoke the release of proinflammatory cytokines, which trigger a pathological series of ACS and/or T2MI since, coronary atherosclerotic plaques comprise different inflammatory cells, which also respond to the systemic proinflammatory cytokines by secretion of local cytokines. These inflammatory reactions induce coronary smooth muscle contraction with subsequent coronary vasospasm.^42^, ^43^ Experimental studies confirm that systemic proinflammatory cytokines, mainly interleukins (IL‐6, IL1, and IL‐8) and TNF‐α, trigger inflammatory cells in the atherosclerotic plaques to secret inflammatory cytokines, which also activate myocardial ischemia through induction of coronary vasospasm.^44^, ^45^, ^46^ In addition, viral respiratory infections may destabilize coronary atherosclerotic plaques through activation of intra‐plaque T cells and macrophages, which induce the release of local coronary peptidase and matrix metalloproteinases (MMPs), which degrade extracellular matrix through induction of oxidative burst.^47^

Moreover, the adaptive immune response is also involved in the development of cardiovascular complications like ACS during respiratory viral infections. Toll‐like receptors 7 (TLR7) are the main innate immune receptors responsible for identifying damage‐associated molecular patterns (DAMPs), which are increased during acute viral infection.^48^ As well, TLR7 is highly expressed in platelets, endothelial cells, and vascular smooth muscle cells. Activation of TLR‐7 induces the release of proinflammatory and anti‐inflammatory cytokines.^48^ In addition, TLR‐7‐activated platelets can increase the risk of thrombus formation by neutrophil aggregation.^48^ Therefore, both adaptive and innate immune responses are activated during respiratory viral infections causing cardiovascular complications including ACS.

**Figure 1:**
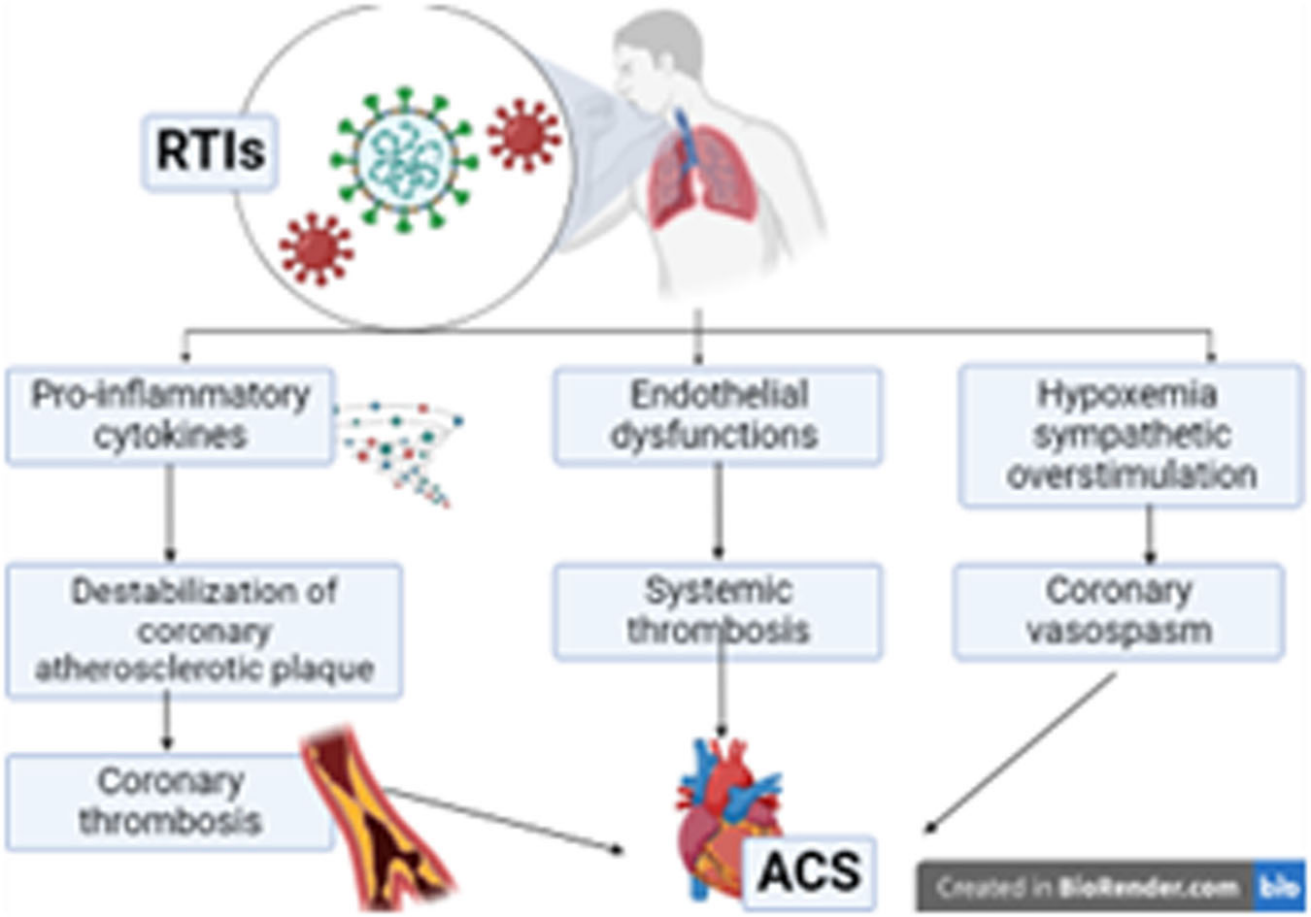
Respiratory tract infections (RTIs) and risk of acute coronary syndrome (ACS): RTIs induce the release of proinflammatory cytokines, which cause destabilization of coronary atherosclerotic plaque and coronary thrombosis. As well, pathogens of RTIs cause endothelial dysfunctions and systemic thrombosis. In addition, RTI may cause hypoxemia and sympathetic overstimulation that leads to coronary vasospasm, these events together leading to the induction of ACS.

Furthermore, corrupted coronary atherosclerotic plaques induce acute coronary thrombosis through the interaction of plaque surface factors, mainly matrix molecules, and phospholipids, with circulating platelets and clotting factors.^49^, ^50^ Likewise, respiratory viral infection directly or indirectly may lead to T1MI through various mechanisms, including platelet activation, inhibition of antithrombin III, suppression of fibrinolytic pathways proteins mainly tissues factor inhibitor and protein C and S that together provoke systemic and coronary thrombotic conditions, leading to ACS and/or T1MI‐related condition^51^ (Figure 1).

It has been reported that bacterial and viral respiratory tract infections are associated with a higher incidence of MI, which might occur during the first week of infection with a respiratory syncytial virus.^52^, ^53^ Notably, 7%–8% of hospitalized patients with pneumococcal pneumonia developed acute T2MI, linked with disease severity.^54^, ^55^ Corrales‐Medina et al.^56^ found that the risk of MI in respiratory tract infection is mainly in the first 15 days following hospitalization due to acute respiratory infection.

Despite clinical entity, differentiation of TIMI from T2MI is difficult at clinical presentation unless percutaneous coronary intervention (PCI) is performed; however, Putot et al.^57^ illustrated that CRP/cTn ratio is higher in T2MI as compared with T1MI, suggesting underlying inflammatory disorders as a causative cause for this type of cardiac ischemia.

These observations suggest that acute respiratory viral infections could be a possible cause for the development and progression of ACS by inducing endothelial dysfunction and dysregulation of immunoinflammatory response.

## 4 COVID‐19 AND RISK OF ACS

In the COVID‐19 era, hospital admission of total ACS cases was reduced dramatically by about 40.7%, mainly in NST‐MI patients that presented later than 24 h of presenting symptoms, which might increase the risk of subsequent complications.^58^ Similarly, a significant reduction in NST‐MI patients' presentation to the emergency department was documented in Hong Kong.^59^ An observational study from a multicenter in Italy observed a significant decline in about 65.4% in NST‐MI patients' hospital admission in 2020 compared to the same duration in 2019. Therefore, ACS‐related complications such as arrhythmias, cardiogenic shock, and septal defects were augmented to 18.18% compared to 10.4% in 2019.^60^ This remarkable reduction in the hospital presentation of ACS cases might be due to substantial and exaggerated afraid from COVID‐19 as well as to ensure the political policy of social distance. Therefore, the death rate outside the hospital and long‐term complications of ACS, mainly MI, were increased with a missed opportunity for secondary prevention.^61^ Rashid et al.^62^ the observational study illustrated that 4% of hospitalized ACS patients, mainly with NST‐MI, were COVID‐19 positive. In the bargain, hospitalized patients with ACS and COVID‐19 showed higher rates of ACS‐related complications such as septic and/or cardiogenic shock with life‐threatening ventricular arrhythmias.^63^, ^64^ Different clinical studies reported the association between SARS‐CoV‐2 infection and ACS. Huang et al.^65^ involved 41 COVID‐19 patients, 13 in ICU and 28 in the general ward, and showed that 31% of COVID‐19 patients in the ICU developed acute MI, with 15% mortality. From Wuhan city, an observational study involved 416 COVID‐19 patients 19.7% developed acute MI consistent with NST‐MI.^66^ A retrospective study by Zhou et al.^67^ revealed that 191 COVID‐19 patients (137) survivors and 54 nonsurvivors), 59% of nonsurvivor, undergo acute MI with a 28.3% general in‐hospital mortality rate. These findings and outcomes proposed that critically ill COVID‐19 patients and nonsurvivors are more prone to develop MI and other aspects of ACS.

COVID‐19‐induced acute MI has been defined as the elevation of high‐sensitivity cTn above the 99th percentile of its upper limit of normal or evidence of new ECG abnormalities.^68^ The presence of increased levels of high‐sensitivity cTn was established to be an independent predictor of disease severity and mortality rate in COVID‐19 even after adjustment for baseline characteristics and medical comorbidities, also showing an association with the need for intensive care unit admission.^68^

The association between SARS‐CoV‐2 infection and progression of ACS and myocardial injury has been confirmed in a cohort observational study involving 2736 COVID‐19 patients; one‐third of them experienced an elevation in cTn serum levels, suggesting myocardial injury. Besides, ECG abnormalities have been shown in about 50% of hospitalized patients.^69^, ^70^ However, permanent cardiac damage or chronic cardiac sequelae of COVID‐19 are not reported following recovery from acute cardiac injury. Nevertheless, SARS‐CoV‐2‐induced heart failure has been reported following acute infection.^71^ Moreover, acute SARS‐CoV‐2 infection is linked with the development of nonobstructive coronary disorders such as coronary vasospasm, dilated cardiomyopathy, myocardial fibrosis, and myocarditis.^72^, ^73^ Acute SARS‐CoV‐2 infection promotes the progression of stable coronary artery disease (CAD) to the severe form of ACS.^74^ However, recovery from COVID‐19 is associated with noteworthy improvement in 12% of cardiac magnetic resonance imaging (MRI).^75^

## 5 MECHANISMS OF COVID‐19‐INDUCED ACS

### 5.1 Acute myocardial injury

The causal relationship between myocardial injury and SARS‐CoV‐2 infection is not yet proven. Even so, reported coronary microthrombi in acute SARS‐CoV‐2 infection have not been uniformly confirmed in cardiac pathology of COVID‐19.^76^, ^77^ Wu et al.^78^ illustrated that SARS‐CoV‐2‐induced myocardial injury is related to different mechanisms, including downregulation of cardiac ACE2, oxidative stress‐induced cardiotoxicity, hypoxemia, and cytokine storm. In addition, Takotsubo syndrome (TTS) is a stress nonischemic cardiomyopathy caused by a sudden weakening of cardiac muscles caused by stressful conditions.^79^ Moderato et al.^80^ reported a case of SARS‐CoV‐2‐induced TTS following the development of T2MI due to a mismatch between myocardial oxygen demand and supply in COVID‐19 respiratory failure. It has been shown that asymptomatic cardiac injury is reported in COVID‐19 patients as evidenced by elevation in cTn serum levels without typical ECG findings of myocarditis.^81^ It has been shown that cardiac involvement in COVID‐19 is through two main pathways; direct through overexpression of ACE2 and indirect through a cytokine storm.^82^ ACE2 is a surface molecule found on vascular endothelial cells, arterial smooth muscle, and cardiac myocytes. When SARS‐COV2 attaches to ACE2 receptors on myocardial cells, it will cause their downregulation. This will result in AngII accumulation and consequently, adverse myocardial remodeling mediated by its action on ACE1 receptors.^83^ Nevertheless, major coronary vessels with atherosclerotic plaques develop minor inflammation during COVID‐19 since coronary arteries are not initially and primarily targeted by SARS‐CoV‐2 due to low expression of ACE2 in coronary vessels.^84^ Also, acute myocardial injury is an independent risk factor for increased mortality in COVID‐19 patients since cTn serum levels in COVID‐19 patients at ICU correlate with high mortality.^85^ In this sense, the SARS‐CoV‐2‐induced release of proinflammatory cytokines and the development of cytokine storms trigger coronary atherosclerotic plaque instability.^86^ Besides, hypoxemia due to ALI/ARDS and respiratory failure, endothelial dysfunction‐induced prothrombotic disorders, and SARS‐CoV‐2‐induced myocarditis could contribute to the development of TIM1 and T2MI.^87^, ^88^

### 5.2 Endothelial dysfunction

COVID‐19 is regarded as a vascular disease leading to endothelial dysfunction and endothelitis that affect various organs due to the binding of SARS‐CoV‐2 to endothelial cells ACE2 with subsequent inflammatory changes.^89^ It has been confirmed that ACE2 and transmembrane protein serine 2 (TMPRSS2) are highly expressed in the heart and vascular endothelial cells. TMPRSS2 cleaves the surface protein (SP) of SARS‐CoV‐2 and increases infectivity and binding of SARS‐CoV‐2 to the ACE2. Endothelial ACE2 is mainly expressed in the epicardial vessels and is nearly few or absent in the coronary vessels.^90^ SARS‐CoV‐2‐induced endothelitis and associated hypercytokinemia lead to intravascular coagulation and thrombotic events, which might impair systemic microcirculation and contribute to the progression of ACS.^91^ Proinflammatory cytokines alter the function of endothelial cells and contribute to thrombosis.^89^ Cytokines including IL‐1β, IL‐6, and TNF‐α the protective functions of the endothelium causing thrombosis. Also, IL‐1 from endothelial cells and invading leukocytes trigger the production of chemoattractant molecules which mediate the penetration of inflammatory cells into tissues. Beun et al.^92^ revealed that COVID‐19‐induced intravascular coagulation is resistant to heparin therapy due to SARS‐CoV‐2‐induced elevation of factor VIII and reduction of antithrombin III. Therefore, the proinflammatory state of COVID‐19 and the consequent endothelial dysfunction may play a role in the progression of ACS.^74^

Therefore, a complex expression of ACE2 in cardiac and small vessels and endothelitis could explain SARS‐CoV‐2‐induced microangiopathy, acute cardiac injury, and development of ACS.

### 5.3 Arrhythmia

COVID‐19‐induced arrhythmia may increase the risk of ACS through the induction of mismatching between oxygen demand and requirement.^93^ The most common arrhythmia related to SARS‐CoV‐2 infection is sinus tachycardia, with palpitations as the principal clinical presentation.^94^ Notably, heart rate in infection is simply a marker of a severe clinical condition and a high systemic response to sepsis at presentation. Though, autonomic dysfunction could be a possible cause of the development of tachycardia in COVID‐19.^95^

Numerous cardiac complications, including new or worsening arrhythmias, are common in pneumonia patients due to COVID‐19.^83^ The pathophysiology of arrhythmia in COVID‐19 could be the result of tissue damage through myocarditis or MI. An extra reason for arrhythmia includes right ventricular strain secondary to pulmonary hypertension or pulmonary embolism.^83^ Moreover, arrhythmia can also be caused by cell‐mediated cytotoxicity by CD8+ T lymphocytes that migrate into the heart and cause myocardial inflammation.^83^ This is mainly driven by the over‐activation of lymphocytes due to cytokine storm resulting in the excessive release of proinflammatory mediators causing a positive feedback loop of immune activation and MI. Other possible mechanisms of arrhythmia are the use of proarrhythmic drugs in the management of COVID‐19, electrolyte imbalances in hospitalized patients, and endogenous catecholamine adrenergic status.^96^ Some recent studies have reported the presence of arrhythmias among patients with COVID‐19. Liu et al.^97^ illustrated that palpitations were reported as the initial symptom in 10 (7.3%) out of 137 COVID‐19 patients presenting to tertiary hospitals in the Hubei province in January 2020. Furthermore, in a global case series conducted in 29 institutions across the world, 827 out of 4526 hospitalized COVID‐19 patients developed an arrhythmia.^98^ The most common of which was atrial fibrillation presenting in 80% of these patients, 20.7% developed ventricular arrhythmias, and 22.6% had bradyarrhythmia.^98^ Furthermore, it was shown that arrhythmias were associated with high morbidity and mortality among those patients: 43% of patients who developed arrhythmia were mechanically ventilated and 51% survived hospital discharge.^98^

Indeed, the persistence of sinus tachycardia and palpitation in subjects suffering from SARS‐CoV‐2 infection has been hypothesized to be related to a long‐term dysregulation of the autonomic system.^95^ Notoriously, SARS‐CoV‐2 infection‐induced release of proinflammatory cytokines induces the blockage of cardiac K channels, and QT prolongation causes malignant ventricular arrhythmias.^99^ Thus, high heart rate at discharge in COVID‐19 patients is not such a frequent problem and it involves 5.5% of the population following COVID‐19. Arrhythmia and tachycardia are robustly related to the evidence of a severe course of COVID‐19. Nevertheless, postdischarge follow‐up data are needed to understand the persistence over time of sinus tachycardia and if this has a prognostic implication or is only the marker of the worst disease.^94^

### 5.4 Rupture of coronary atherosclerotic plaque

It has been reported that atherosclerosis, mainly atherosclerotic plaque, is considered an inflammatory process as associated dyslipidemia and hypertension increase endothelial vascular wall permeability for accumulation of inflammatory cells, chiefly lymphocytes and monocytes, into the subendothelial area.^100^ In turn, these inflammatory cells provoke oxidation, expression of vascular adhesion molecule 1 (VCAM‐1), and infiltration of T cells with the generation of foam cells and constant inflammatory process.^101^ Therefore, the persistent inflammatory response is prone to building plaque to be a vulnerable plaque, which is susceptible to erosion and rupture with risk of thrombosis and development of complications as revealed in acute exacerbation of ACS during sepsis.^102^

SARS‐CoV‐2 infection can induce rupture of coronary atherosclerotic plaque by different mechanisms including endothelial injury, the release of proteolytic enzymes, and erosion of plaque cap by inducing the release of matrix metalloproteinase 9 (MMP‐9).^103^, ^104^ Development of cytokine storm in severe SARS‐CoV‐2 infection promotes expression and release of MMP‐9 which is implicated in the erosion and rupture of coronary atherosclerotic plaque.^105^ A previous prospective study involved patients with ACS showed that Ang II, AT_1_ receptor, and ACE are highly expressed in human atherosclerotic coronary arteries.^106^ This finding suggests that Ang II is produced primarily by ACE within coronary plaques. The observation that Ang II induces IL‐6 and their colocalization with the AT_1_ receptor and ACE is consistent with the notion that the RAS may contribute to inflammatory processes within the vascular wall and to the development of ACS. Of note, AngII is exaggerated in severe SARS‐CoV‐2 infection^107^ and this may explain the higher incidence of ACS in COVID‐19. These observations indicated that cytokine storm and dysregulation of RAS might be a possible mechanism for the development of ACS in patients with severe COVID‐19.

## 6 DISCUSSION

### 6.1 COVID‐19 and ACS

In particular, ACS is associated with systemic inflammatory disorders as evidenced by high CRP, IL‐6, and procalcitonin serum levels as well as neutrophils, macrophages, and T lymphocytes in the coronary bed.^108^ Polidoro et al.^109^ confirmed that SARS‐CoV‐2 infection is linked with systemic inflammatory reactions, as evidenced by high circulating levels of CRP, IL‐6, and procalcitonin that might affect coronary atherosclerotic plaque stability augmentation of cardiac preload and afterload. Both CRP and IL‐6 are regarded as independent risk factors and correlated with the severity of ACS.^110^ In SARS‐CoV‐2 infection, nuclear factor kappa B cell (NF‐κB) signaling and nod‐like receptor pyrin 3 (NLRP3) inflammasome are activated directly by SARS‐CoV‐2 viral proteins leading to inflammatory burst through induction release of proinflammatory cytokines.^111^, ^112^ NLRP3 inflammasome is involved in the inflammatory process to develop atherosclerosis and coronary atherosclerotic plaque formation and destabilization.^113^ Mauro et al.^114^ found that activation of NLRP3 inflammasome is linked with the development of acute MI. Besides, a recent study illustrated that activation of NLRP3 inflammasome might cause acute cardiomyocyte injury.^115^

On the other hand, activation of NF‐κB signaling is connected with the development of coronary microembolization and acute cardiac injury.^116^, ^117^ These findings support the proposed theory that SARS‐CoV‐2 infection through activation of NLRP3 inflammasome and NF‐κB signaling may lead to T2MI in critically COVID‐19 patients. However, Cremer^118^ illustrated that little is known about SARS‐CoV‐2 infection through the hyperinflammatory state to cause either T1MI or T2MI despite known direct cardiomyocyte injury. In line with advanced research, Newby et al.^119^ showed that the mitogen‐activated protein kinase (MAPK) level is associated with acute MI in patients with ACS. MAPK signaling is highly activated during SARS‐CoV‐2 infection and is involved in the acute inflammatory response and cardiomyocyte injury. MAPK signaling inhibitor is also a promising therapeutic target in managing acute SARS‐CoV‐2 infection.^120^ These verdicts suggest that SARS‐CoV‐2 infection‐induced activation of MAPK signaling might be a possible pathway in developing new‐onset ACS in severely affected COVID‐19 patients.

Of note, high Ang II in COVID‐19 due to downregulation of ACE2 by SARS‐CoV‐2 SP may cause acute cardiomyocyte injury and coronary vasoconstriction.^121^, ^122^ Therefore, angiotensin‐converting enzyme inhibitors (ACEIs) and angiotensin receptor blockers (ARBs) may mitigate and reduce the risk of ACS and MI through the upregulation of cardioprotective ACE2 with the regulation of the balance of Ang II/Ang‐17 axis, which prevents inflammation and thrombosis.^123^ Furthermore, various studies illustrated that CD147, a molecule of inflammation and proteolysis, is involved in the progression and pathogenesis of ACS as high circulating plasma CD147 is linked with ACS severity.^124^ In addition, CD147 plasma level is positively correlated with cTn serum level in patients with ACS.^125^ Helal et al.^126^ in a silico study illustrated that the binding of SARS‐CoV‐2 SP with CD147 led to the activation of inflammation and induction of lymphopenia. Furthermore, both CD147 and lymphopenia are correlated with the rupture of coronary plaques.^126^, ^127^ However, a recent in vitro study confirmed no role for CD147 as a receptor for SARS‐CoV‐2 since the ablation of this receptor did not affect the infectivity of this virus.^128^

**Figure 2:**
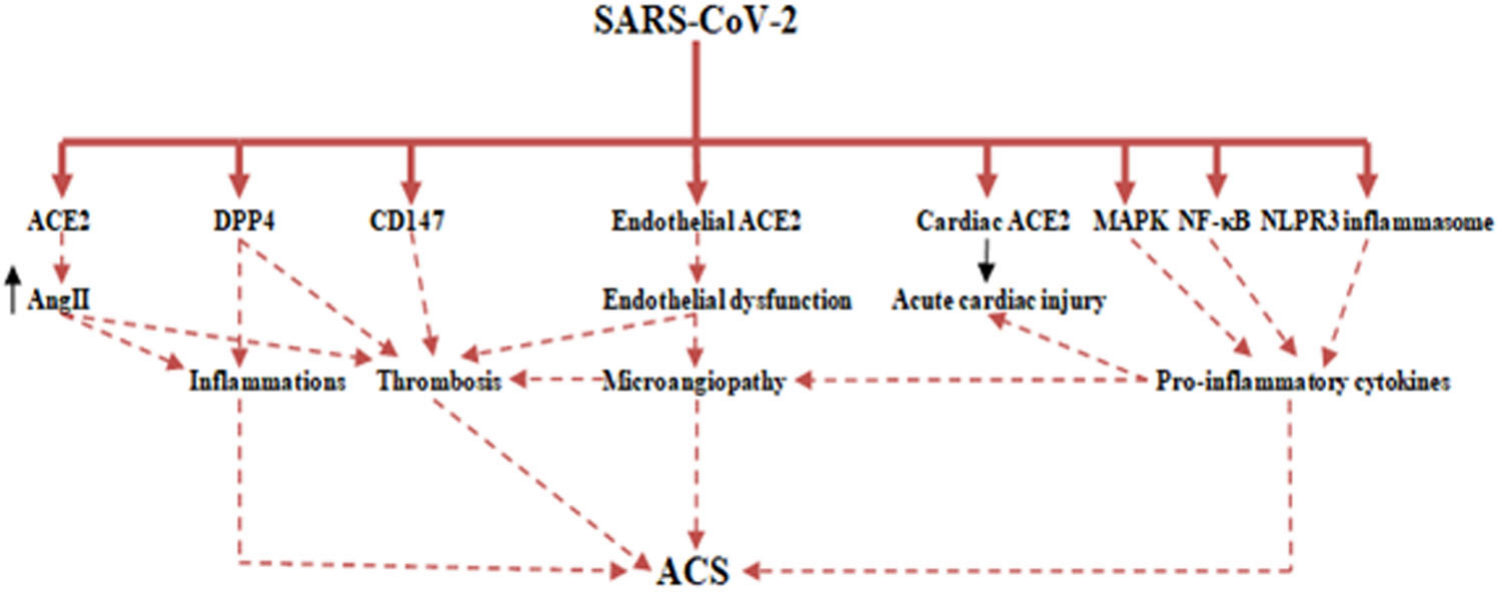
Mechanism of SARS‐CoV‐2‐induced acute coronary syndrome (ACS): SARS‐CoV‐2 through binding angiotensin‐converting enzyme 2 (ACE2) increases circulating angiotensin II (Ang II) and through dipeptidyl peptidase 4 (DPP4) and CD147 induces inflammation and thrombosis. Downregulation of endothelial ACE2 by SARS‐CoV‐2 leads to endothelial dysfunction and microangiopathy. In addition, SARS‐CoV‐2 induces activation of mitogen‐activated protein kinase (MAPK), nuclear factor‐kappa B cell (NF‐κB) signaling, and nod‐like receptor pyrin 3 (NLRP3) inflammasome leading to the release of proinflammatory cytokines, which cause acute cardiac injury and microangiopathy. Taken together, these changes provoke ACS.

Furthermore, dipeptidyl peptidase 4 (DPP4) has an inflammatory role in the pathogenesis of atherosclerosis and ACS therefore, DPP4 inhibitors such as sitagliptin reduce the severity of ACS in diabetic patients. In addition, DPP4 promotes the progression of coronary atherosclerotic plaques by enhancing monocyte migration and inhibiting the protective endothelial signaling pathway of the glucagon‐like peptide.^129^ Recently, DPP4 is a potential receptor for entry of SARS‐CoV‐2, and DPP4 inhibitors might be a potential therapeutic strategy in treating COVID‐19 even in nondiabetic patients^130^, ^131^ (Figure 2).

Taken together, there is an intricate relationship between SARS‐CoV‐2 infection and associated inflammatory response with complications of ACS due to erosion/rupture of coronary atherosclerotic plaque.^132^, ^133^ As mentioned earlier, COVID‐19‐induced hypoxia by ARDS, SARS‐CoV‐2‐induced cardiomyocyte injury, cardiomyocyte interstitial injury, and cytokine storm may together affect coronary microcirculation. These changes lead to the induction of T2MI through‐provoking release of the clotting factor and von‐Willebrand factor with inhibition of endothelial anticoagulant activity. These interactions activate a continuous procoagulant and proinflammatory state that predispose to systemic thrombosis.^134^ Yet, microcoronary thrombosis was not documented in COVID‐19 patients. However, systemic inflammatory condition that may cause coronary vasculitis is evident in Kawasaki‐like disease in COVID‐19 children.^135^ Sokolovsky et al.^136^ reported a case reported of Kawasaki‐like disease in an adult COVID‐19 patient. It is well‐known that Kawasaki disease leads to ACS and new‐onset T2MI in children and adults.^137^ Therefore, a systemic hyperinflammatory state mimics Kawasaki disease that might cause ACS in critically COVID‐19 patients. Thus, direct myocardial injury by SARS‐CoV‐2 or indirect injury by cytokine storm with the development of cardiac arrhythmia^94^ may increase the risk for the development of MI in COVID‐19 patients.

Taken together, PCI and serial cTn assessment are mandatory in critically COVID‐19 patients to confirm the underlying causes of ACS, which are either occlusive or nonocclusive, and to observe types of MI associated with COVID‐19. Therefore, retrospective and large‐scale prospective studies are reasonable in this regard.

### 6.2 Anti‐COVID‐19 medications and ACS

Chloroquine and hydroxychloroquine are initially used to manage COVID‐19 depending on the in vitro findings. However, most clinical trials did not prove the efficacy of these drugs in treating COVID‐19 and revealed significant cardiotoxicity, so in June 2020 FDA re‐evaluated the clinical efficacy in the management of COVID‐19.^138^ A meta‐analysis by Axfors et al.^139^ found that chloroquine has no benefit in treating COVID‐19, while hydroxychloroquine treatment in COVID‐19 is associated with high mortality. Furthermore, Monzani et al.^140^ illustrated that hydroxychloroquine treatment in COVID‐19 is associated with QT prolongation due to blocking cardiac Na^2+^, which causes sudden cardiac death. In addition, chloroquine therapy may lead to cardiotoxicity even at a low dose and cause cardiogenic shock in susceptible patients.^141^, ^142^ Therefore, chloroquine or hydroxychloroquine‐induced arrhythmia may cause mismatching between cardiomyocyte oxygen demand and supply leading to induction of ACS in COVID‐19 patients.^143^ In addition, treatment of patients with COVID‐19 with chloroquine and hydroxychloroquine is associated with an important risk of drug‐induced QT prolongation and relatively higher incidence of torsades de pointes, ventricular tachycardia, or cardiac arrest.^144^ Therefore, these agents should not be used routinely in the management of COVID‐19 disease. Patients with COVID‐19 who are treated with antimalarial agents for other indications should be adequately monitored. However, long‐term hydroxychloroquine therapy decreases the risk of cardiovascular complications in systemic lupus erythematosus patients. The cardiovascular protective effect of hydroxychloroquine therapy was associated with a decrease in coronary artery disease.^145^ As well, a systematic review and meta‐analysis showed that hydroxychloroquine substantially reduces cardiac mortality and also decreases thrombosis, arrhythmia, and cholesterol in treated patients in recent peer‐reviewed studies and meeting presentations.^146^ Hydroxychloroquine in combination with azithromycin does not cause torsades de pointes cardiac mortality; rather, hydroxychloroquine decreases cardiac events. Therefore, hydroxychloroquine should not be restricted in COVID‐19 patients out of fear of cardiac mortality.^146^ These findings proposed that the use of chloroquine and hydroxychloroquine seems to be protective against the development of MI in COVID‐19 patients.

Lopinavir and ritonavir are antiviral agents used to manage mild‐moderate COVID‐19 and have been shown to cause QT prolongation and cardiac arrhythmia by blocking cardiomyocyte potassium current channels, mainly when combined with hydroxychloroquine.^147^ In addition, a combination of lopinavir and ritonavir known under the brand name Kaletra is subjected to drug interaction through inhibition of CYP3A and P‐glycoprotein, leading to ventricular arrhythmia. Also, this combination leads to dyslipidemia that may aggravate underlying ACS.^148^ Lopinavir/ritonavir, in combination with chloroquine and hydroxychloroquine, may cause cardiotoxicity by acting on cardiac potassium channels, especially the hERG channel through their off‐target effects. The blocking of the hERG channel prolongs QT intervals on ECG; thus, it might induce severe ventricular arrhythmias and even sudden cardiac death.^149^ Notably, long‐term use of lopinavir/ritonavir in HIV‐1 patients was not associated with an increased risk for MI.^150^

Antibiotic azithromycin was elected to be effective against SARS‐CoV‐2 infection and is regarded as a potential proarrhythmogenic agent associated with cardiovascular death through QT prolongation.^151^ Azithromycin in combination with hydroxychloroquine increase the risk of developing ventricular arrhythmia as both drugs have torsadogenic potential.^152^ A meta‐analysis and systematic review by Diaz‐Arocutipa et al.^153^ illustrated that although the combination of azithromycin with hydroxychloroquine is linked with a high risk of QT prolongation in COVID‐19, the prevalence of associated arrhythmia is very low due to underreporting of relevant cases.

Remdesivir, a broad‐spectrum antiviral drug initially used to treat hepatitis C, is approved for the treatment of COVID‐19. Remdesivir is less subjected to the metabolism by P450 enzymes, and its parenteral administration is associated with severe allergic reactions.^154^ In addition, hydroxychloroquine reduces the antiviral effect of remdesivir through inhibition of its intracellular activation.^155^ Although in most recent studies concerning the role of remdesivir in COVID‐19, few studies reported the proarrhythmogenic effect of remdesivir, Barkas et al.^156^ revealed that remdesivir might cause severe bradycardia in critically COVID‐19 patients.

Therefore, anti‐COVID‐19 medications through induction of arrhythmias and cardiotoxicity may affect coronary blood flow and myocardial oxygenation with increased risk for the development of MI in severely affected COVID‐19 patients. This finding needs to be confirmed by preclinical and large‐scale clinical studies.

Taken together, ACS and T2MI in COVID‐19 patients, according to the evidence from published studies, are mainly related to the effect of SARS‐CoV‐2‐induced hypercytokinemia and direct acute cardiac injury rather than to arrhythmias induced by anti‐COVID‐19 medications.

## 7 CONCLUSION

SARS‐CoV‐2 infection through hypercytokinemia, direct cardiomyocyte injury, and dysregulation of the RAS may aggravate underlying ACS or cause new‐onset T2MI. As well, arrhythmias induced by anti‐COVID‐19 medications could worsen underlying ACS. Despite these findings, we cannot give from this review a definitive conclusion regarding the role of COVID‐19 in the pathogenesis of ACS. Large retrospective and prospective studies are necessary in this regard to justify these findings.

## AUTHOR CONTRIBUTIONS

**Aseel Awad Alsaidan**: Writing—review & editing. **Hayder M. Al‐Kuraishy**: Conceptualization; Writing—original draft. **Ali I. Al‐Gareeb**: Conceptualization; Writing—original draft. **Athanasios Alexiou**: Resources; Writing – review & editing. **Marios Papadakis**: Writing—review & editing. **Khalid Adel Alsayed**: Writing—review & editing. **Hebatallah M. Saad**: Supervision; Writing— review & editing. **Gaber El‐Saber Batiha**: Validation; Writing—review & editing.

## CONFLICT OF INTEREST STATEMENT

The authors declare no conflict of interest.

## ETHICS STATEMENT

Not applicable.

## Data Availability

Not applicable.
